# Soil biota in vineyards are more influenced by plants and soil quality than by tillage intensity or the surrounding landscape

**DOI:** 10.1038/s41598-017-17601-w

**Published:** 2017-12-12

**Authors:** Jacob Buchholz, Pascal Querner, Daniel Paredes, Thomas Bauer, Peter Strauss, Muriel Guernion, Jennifer Scimia, Daniel Cluzeau, Françoise Burel, Sophie Kratschmer, Silvia Winter, Martin Potthoff, Johann G. Zaller

**Affiliations:** 1 Institute of Zoology, University of Natural Resources and Life Sciences Vienna (BOKU), Gregor Mendel Straße 33, 1180 Vienna, Austria; 2 Grupo de Protección Vegetal, Departamento de Protección Ambiental, Estación Experimental de Zaidín, CSIC, Profesor Albareda n◦ 1, 18008 Granada, Spain; 3 Institute for Land and Water Management Research, Austrian Federal Agency for Water Management, Pollnbergstraße 1, A-3252 Petzenkirchen, Austria; 4 Université de Rennes I, OSUR, UMR CNRS 6553 ‘EcoBio’, Station Biologique de Paimpont, 35380 Paimpont, France; 5 Université de Rennes I, OSUR, UMR CNRS 6553 ‘EcoBio’, Avenue du Général Leclerc Campus de Beaulieu, F-35042 Rennes Cedex, France; 6 Institute of Integrative Nature Conservation Research, University of Natural Resources and Life Sciences Vienna, Gregor Mendel Straße 33, 1180 Vienna, Austria; 7 Centre of Biodiversity and Sustainable Land Use (CBL), University of Göttingen, Grisebachstraße 6, 37077 Göttingen, Germany

## Abstract

Tillage is known for its adverse effects on soil biota, at least in arable agroecosystems. However, in vineyards effects might differ as tillage is often performed during dry periods or only in every other inter-row allowing species to re-colonise disturbed areas. We examined the response of earthworms (lumbricids), springtails (collembola) and litter decomposition to periodically mechanically disturbed (PMD) and permanently green covered (PGC) vineyard inter-rows and assessed whether site effects are altered by the surrounding landscape. In commercial vineyards in Austria we sampled earthworms by handsorting, springtails by soil coring and pitfall trapping and installed litter decomposition bags. Earthworm species diversity increased with plant biomass under PMD but not under PGC; earthworm density was unaffected by tillage but increased with plant biomass mainly at high soil quality (soil fertility index). Springtail species diversity was unaffected by tillage; springtail densities (mainly larger species) were reduced under PGC. Litter decomposition was little affected by investigated parameters. Landscape heterogeneity affected the functional diversity of surface springtails, but did not influence soil-dwelling springtails, earthworms or litter decomposition. We conclude that effects on soil biota of periodical tillage in vineyards need not necessarily be detrimental and will be modified by plant biomass and soil quality.

## Introduction

Viticulture is an ancient and globally widespread agricultural production system. Smallholder wine-growers contribute to multifunctional landscapes that, in addition to grape and wine production, provide a variety of ecosystem services^1^. Vineyards are often intensively managed involving frequent tilling and/or pesticide treatments^2^. Inter-row soil management practices are performed for weed control, water conservation or to prevent erosion. These soil tillage regimes include permanent bare soil as a result of intensive tillage, moderate tillage of every second inter-row only (also called alternating tillage) or no tillage, leaving permanently vegetated inter-rows^3^. Soil tillage has been shown to negatively affect soil biodiversity which is directly linked to associated ecosystem functions such as nutrient cycling or soil formation^4^. The compostion and biomass of vegetation may also modify the food source for soil organisms thereby altering their community composition^5,6^. However, only a few studies have investigated effects of vineyard soil management practices on earthworms^7–11^, springtails^12,13^ or soil microbial communities^14,15^. These studies mainly compared very contrasting management measures such as intensive tillage, or herbicide applications vs. permanent vegetation cover. To the best of our knowledge nothing is known on the effects on soil biota in vineyards with alternating tillage vs. no tillage.

In vineyards, as in many other agroecosystems, the role of earthworms (Oligochaeta: Lumbricidae) in promoting soil fertility, aggregate formation and soil organic matter protection is important^16^. Generally, earthworms are sensitive to soil tillage^17^, and have been used as bioindicators for sustainable soil use and soil quality^18–20^. Earthworms can be divided into three ecological groups according to their habitat and functional role in the agroecosystem^21^. Epigeic species live in the top soil and litter layer and usually do not build permanent burrows. Anecic species drill vertical burrows reaching from the soil surface down to subsoil layers. Endogeic species burrow mainly horizontally in the upper mineral soil layers. In vineyards, earthworm abundance has been shown to reach 241 worms m^−2^, but this can vary considerably^8^. Other important indicators of soil quality and sustainable land use are springtails (Hexapoda: Collembola^22–27^). By feeding on fungi, bacteria and dead organic matter, as well as living plant tissue they mobilize locked-up nutrients^28,29^, contribute to supporting ecosystem services, and are sensitive to land use changes^30,31^. Springtails are among the most abundant microarthropods in soil^32,33^, with densities of up to 60.000 ind. m^−2^ in grasslands^34^. Springtails can be grouped into the three life forms: eu- and hemiedaphic species live mainly in the soils while epedaphic species are active on the soil surface^32,35^. Earthworm and springtail community changes are considered to be powerful indicators for ecosystem service provision in vineyard soils. Another important process integrating the activity of soil biota is litter decomposition^36^. For example, mesofaunal contribution to litter decomposition is directly involved in the degradation of material and indirectly by feeding on microorganisms. Microorganisms in soil have also been shown to be sensitive to tillage practices^37^. Yet, also other practices such as pesticide applications can modify litter decomposition rates^38,39^.

There is a growing body of studies showing that both plant and aboveground animal biodiversity in vineyards are influenced by the diversity and structure of the surrounding landscape^40–45^. For example, plant communities in vineyards have been shown to be positively affected by nearby semi-natural habitats^43^, specialized ambush spiders in vineyards increased with landscape heterogeneity based on land use categories^40^, sedentary butterfly species in vineyards were related to remnant patches of native vegetation in the surrounding landscape^41^. We also know from studies in arable crops that both earthworms and springtails were affected by the surrounding landscape structure and land-use intensity within a radius of several hundred meters^46–52^. However, to the best of our knowledge it has never been examined whether soil biota inhabiting vineyards are also influenced by the surrounding landscape structure.

The aim of this study was to examine the response of earthworm and springtail communities and litter decomposition to periodically mechanically-disturbed (PMD) or permanently green covered (PGC) inter-row management in vineyards and whether this response is influenced by the surrounding landscape structure. We expected that (i) earthworms will be more vulnerable to soil disturbance than springtails due to their body size, (ii) both earthworms and springtails will be positively related to plant diversity measures and (iii) landscape complexity will alter potential effects of springtails or earthworms. These interactions were tested in 16 viticultural landscapes in Austria.

## Results

### Earthworms

In our vineyards we found a total of 564 individuals of earthworms, comprising 13 species (5 anecic and 8 endogeic spp.; a full species list is provided in Supplementary Table S1). The mean earthworm density was 146 ± 132 ind. m^−2^ (mean ± sd) across all samples; the maximum of 550 ind. m^−2^ and 286 g m^−2^ was reached in a PMD inter-row (Supplementary Table S1). Overall, 83% of earthworms were endogeics (most abundant spp. was *Aporrectodea caliginosa*), 17% belonged to anecics (most abundant spp was *Lumbricus terrestris*); no epigeics were found.

**Figure 1:**
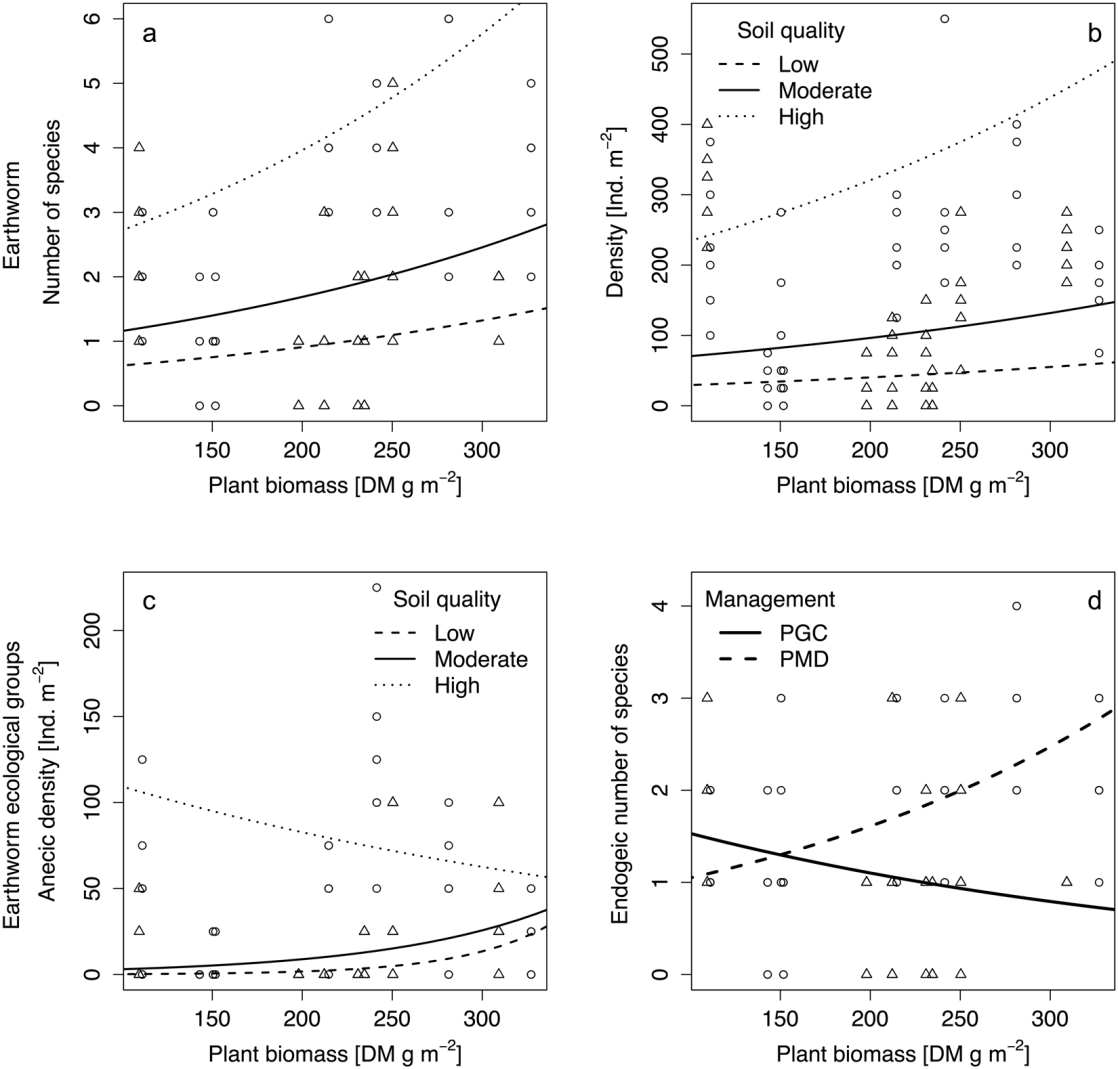
Total earthworm species numbers (**a**) and density (**b**), anecic density (**c**) and endogeic species numbers (**d**) in vineyard inter-rows in response to plant biomass, soil quality and soil inter-row management (PGC…permanent green cover, PMD…periodical mechanical disturbance). O…PGC, Δ…PMD, N = 6.

Earthworm species richness, density and biomass were best explained by soil quality (expressed by the soil field index) and plant biomass (richness: R^2^_m_ = 0.27; density: R^2^_m_ = 0.30; biomass: R^2^_m_ = 0.26; Table 1). Both soil quality and plant biomass increased the number of earthworm species and earthworm density (Fig. 1a,b). Similar patterns were observed for earthworm biomass, however data are not shown. Lowest species richness of earthworms was predicted for vineyards with low soil quality (field index 23), and highest earthworm species richness for vineyards with a high soil quality (field index 67; Fig. 1a). The effect of plant biomass on earthworms was small in sites with low soil quality, but increased with increasing soil quality (Fig. 1a). Similar patterns were found for earthworm density (Fig. 1b), with the lowest density in vineyards with low soil quality, and highest density in vineyards with high soil quality. Again, the effect of plant biomass on earthworm density was higher in sites with high soil quality.

**Table 1:** Comparison of alternative models (using AICc) for earthworms (richness...spp. m^−2^, density...ind. m^−2^, biomass...g m^−2^) and litter decomposition (S value…stabilisation index, k…decomposition rate) in vineyards.

Fixed effect	Random effect	Earthworms, total			Anecic Earthworms		Endogeic Earthworms		Litter decompostion	
		Richness	Density	Biomass	Richness	Density	Richness	Density	S value	k value
No	Yes	314.0	510.0	477.0	163.6	230.4	263.0	445.8	−134.1	126.0
Management intensity	Yes	311.6	510.8	476.2	162.9	230.0	260.5	446.5	−132.3	127.8
Soil quality	Yes	312.4	510.1	476.3	163.0	229.4	261.3	445.8	−133.0	126.7
Managem. intens. × Plant biomass	No	304.2	713.2	489.6	161.9	278.6	**243.7**	569.5	−142.0	117.7
Soil quality + Plant biomass	No	297.2	669.1	474.7	155.8	241.0	**244.5**	571.6	−139.6	116.4
Soil quality × Plant biomass	No	299.4	671.0	477.0	**153.3**	235.9	245.9	573.7	−137.7	117.0
Plant biomass	Yes	300.3	496.0	451.4	160.5	227.9	251.5	433.1	**−157.3**	**112.0**
Managem. intens. + Plant biomass	Yes	297.6	497.4	450.6	159.8	227.7	249.2	434.4	**−155.5**	**114.0**
Managem. intens. × Plant biomass	Yes	298.1	498.6	452.9	161.7	229.9	246.0	435.0	**−155.5**	115.3
Soil quality + Plant biomass	Yes	**293.7**	**493.0**	**447.0**	156.7	**223.1**	246.4	**430.5**	**−155.7**	**112.9**
Soil quality × Plant biomass	Yes	295.9	495.1	449.3	155.5	**223.6**	247.9	432.7	−153.6	114.6
Mean patch size (MPS)	Yes	315.4	509.2	475.9	164.9	231.4	264.2	444.8	−132.0	128.2
Managem. intens. + MPS	Yes	313.1	510.1	475.0	164.1	230.8	261.8	445.5	−130.2	130.0
Managem. intens. × MPS	Yes	312.9	510.1	476.5	159.4	227.4	263.5	446.4	−128.2	131.7
Soil quality + MPS	Yes	313.2	507.9	472.3	163.9	229.7	261.8	443.1	−130.8	128.8
Soil quality × MPS	Yes	313.9	509.4	474.4	166.0	231.8	261.4	444.8	−131.5	130.9
R^2^ adjusted					0.29		0.31			
R^2^marginal		0.27	0.30	0.26		0.33		0.22	0.08	0.06
R^2^conditional		0.41	0.79	0.55		0.60		0.75	0.26	0.19

The best models (lowest AICc and ∆AICc ≤ 2) are indicated in boldface type. R^2^ refers to the best models. For clarity, only the most parsimonious (i.e., lowest AICc) of all possible models are presented. Random effect indicates whether the individual vineyard was included or not.

Response of anecic earthworm density was best explained by soil quality, plant biomass and the interaction between those two variables (R^2^_m_ = 0.33; Table 1). On sites with low soil quality, fewer anecics were found when plant biomass was low (Fig. 1c). Anecic density started to increase above 180 g m^−2^ plant biomass and reached a maximum with plant biomass of 350 g m^−2^. On sites with high soil quality, highest anecic densities (57 ind. m^−2^) were found with low inter-row plant biomass, and decreased with increasing plant biomass.

Endogeic species richness was best explained by soil management, plant biomass, interaction between management and plant biomass and soil quality, (R^2^_adj_ = 0.31; Table 1). Under PGC management the effect of plant biomass on the number of endogeic species was negative (Fig. 1d). However, under PMD effect of plant biomass was positive and higher with a threefold increase in the species numbers along the plant biomass gradient. Responses of endogeic species richness and density to plant biomass and soil quality was similar to overall earthworm richness and density (R^2^_m_ = 0.22).

Neither anecic nor endogeic earthworms were affected by the surrounding landscape.

### Springtails

We collected 12784 ind. of springtails comprising 60 different species. Mean density of the soil dwelling springtails was 37315 ± 35322 ind. m^−2^ (most abundant spp. *Parisotoma notabilis*) and mean activity density of surface dwelling springtails was 70 ± 52 ind. pitfall sample^−1^ (most abundant spp. *Isotoma viridis*; a full species list is provided in Supplementary Table S2).

**Figure 2:**
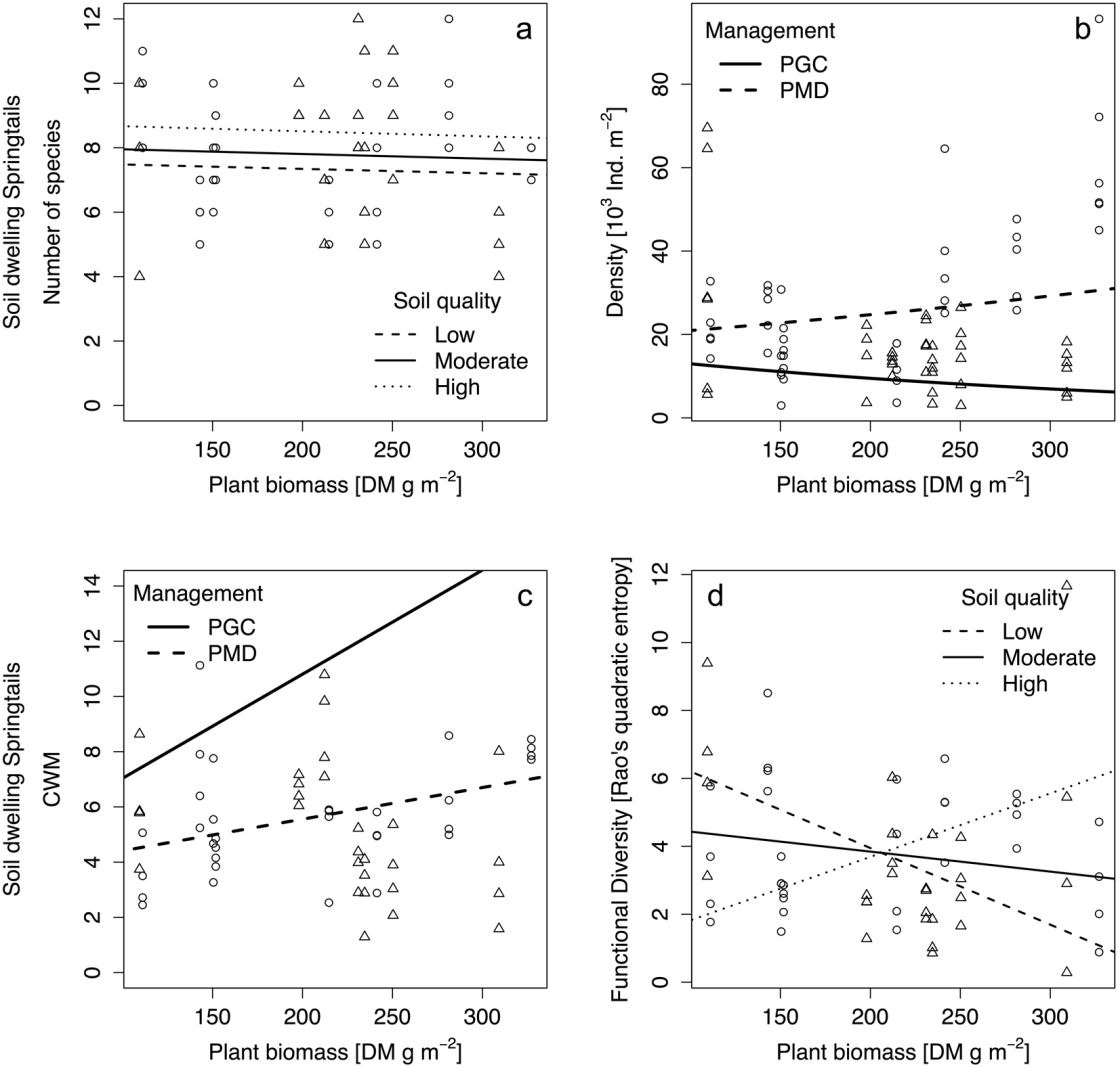
Soil springtail species numbers (**a**), density (**b**), community weighted morphological trait value (CWM, (**c**) and functional diversity (**d**) in response to plant biomass, soil quality and inter-row soil management (PGC…permanent green cover, PMD…periodical mechanical disturbance. O…PGC, Δ…PMD, N = 4.

Soil-dwelling springtail species richness was affected by plant biomass and soil quality (R^2^_adj_ = 0.32; Table 2; Fig. 2a). The effect of plant biomass on springtail species richness was low with a slight decrease of springtail species numbers with increasing plant biomass. Soil quality effect on springtail species numbers was very small, however, a positive effect was seen along an increasing gradient of soil quality.

**Table 2:** Comparison of alternative models (using AICc) for springtails in vineyards (richness...spp. m^−2^, density...ind. m^−2^, CWM...community weighted morphological trait value, FD...functional diversity Rao’s quadratic entropy).

Fixed effect	Random effect	Soil dwelling springtails				Surfaces dwelling springtails			
		Richness	Density	CWM	FD	Richness	Density	CWM	FD
No	Yes	289.2	695.7	273.6	275.3	413.1	884.3	414.5	172.4
Management intensity	Yes	291.4	697.7	275.7	277.3	415.2	884.3	416.2	174.0
Soil quality	Yes	291.3	698.0	275.8	277.6	413.7	884.5	413.5	170.4
Plant biomass	No	**267.4**	693.4	271.9	269.2	**378.7**	850.7	445.8	167.0
Management intensity + Plant biomass	No	269.4	736.4	273.6	271.2	**377.7**	886.0	447.1	162.9
Management intensity × Plant biomass	No	271.1	693.4	266.9	272.7	**377.9**	**810.9**	443.4	165.0
Soil quality + Plant biomass	No	**268.8**	692.7	273.9	271.5	380.1	849.8	434.4	163.7
Soil quality × Plant biomass	No	271.1	695.0	267.2	**267.1**	381.5	841.3	431.9	163.9
Plant biomass	Yes	269.6	**661.6**	**258.1**	**266.2**	380.0	823.6	**393.1**	161.6
Management intensity + Plant biomass	Yes	271.7	**663.2**	260.2	268.4	379.8	822.3	395.1	160.8
Management intensity × Plant biomass	Yes	273.5	663.6	**258.7**	270.4	380.2	**811.9**	395.9	163.1
Soil quality + Plant biomass	Yes	271.1	664.0	260.3	268.6	381.6	824.2	**391.7**	160.9
Soil quality × Plant biomass	Yes	273.4	666.3	**258.8**	**267.0**	383.2	822.4	**392.7**	162.0
Mean patch size (MPS)	Yes	289.4	696.4	275.4	274.5	404.0	885.3	416.6	162.1
Management intensity + MPS	Yes	291.6	698.6	277.5	276.7	405.7	884.8	418.3	162.2
Management intensity × MPS	Yes	293.7	699.6	279.6	278.6	407.4	886.6	420.6	164.5
Soil quality + MPS	Yes	291.4	698.7	277.5	276.9	405.4	885.9	415.7	**159.6**
Soil quality × MPS	Yes	281.5	682.1	279.9	273.3	407.2	888.2	417.8	161.9
R^2^ adjusted		0.32	NA			0.43	NA		
R^2^marginal				0.15	0.12			0.26	0.31
R^2^conditional				0.48	0.28			0.71	0.34

The best models (lowest AICc and ∆AICc ≤ 2) are indicated in boldface type. R^2^ refer to the best models. For clarity, only the most parsimonious (i.e., lowest AICc) of all possible models are presented. Random effect indicates whether the individual vineyard was included or not. CWM… community weighted morphological trait value, FD…functional diversity.

Soil-dwelling springtail density was best explained by soil management and plant biomass (R^2^_adj_ = NA; Table 2; Fig. 2b). Springtail density was noticeable higher in vineyards with PMD, compared to PGC. Under PGC springtail density decreased with increasing plant biomass, while under PMD springtail density increased with increasing plant biomass. The dominant morphological trait value in the springtail community (CWM) was best explained by plant biomass and plant biomass interacting with management and soil quality (R^2^_m_ = 0.15; Table 2). In vineyards with PGC the CWM was higher compared to vineyards under PMD and increased more than double when plant biomass increased (Fig. 2c). Under PMD, CWM increased almost twofold with increasing plant biomass. There was a strong interaction between plant biomass and the soil quality. On sites with low soil quality, plant biomass noticeably reduced springtail CWM, while in moderate soil qualities no relationship between CWM and plant biomass was seen. By contrast, on sites with high soil quality, CWM increased with increasing plant biomass.

Functional diversity of soil-dwelling springtails was affected by plant biomass, soil quality and their interaction (R^2^_m_ = 0.12; Table 2; Fig. 2d). Similarly to the response of springtail CWM to plant biomass and soil quality, plant biomass adversely effected springtail functional diversity when soil quality was low. However, at sites with high soil quality, functional diversity of springtails increased notably along a gradient of increasing plant biomass. When soil quality was moderate, plant biomass showed a slighty negative effect on functional diversity

**Figure 3:**
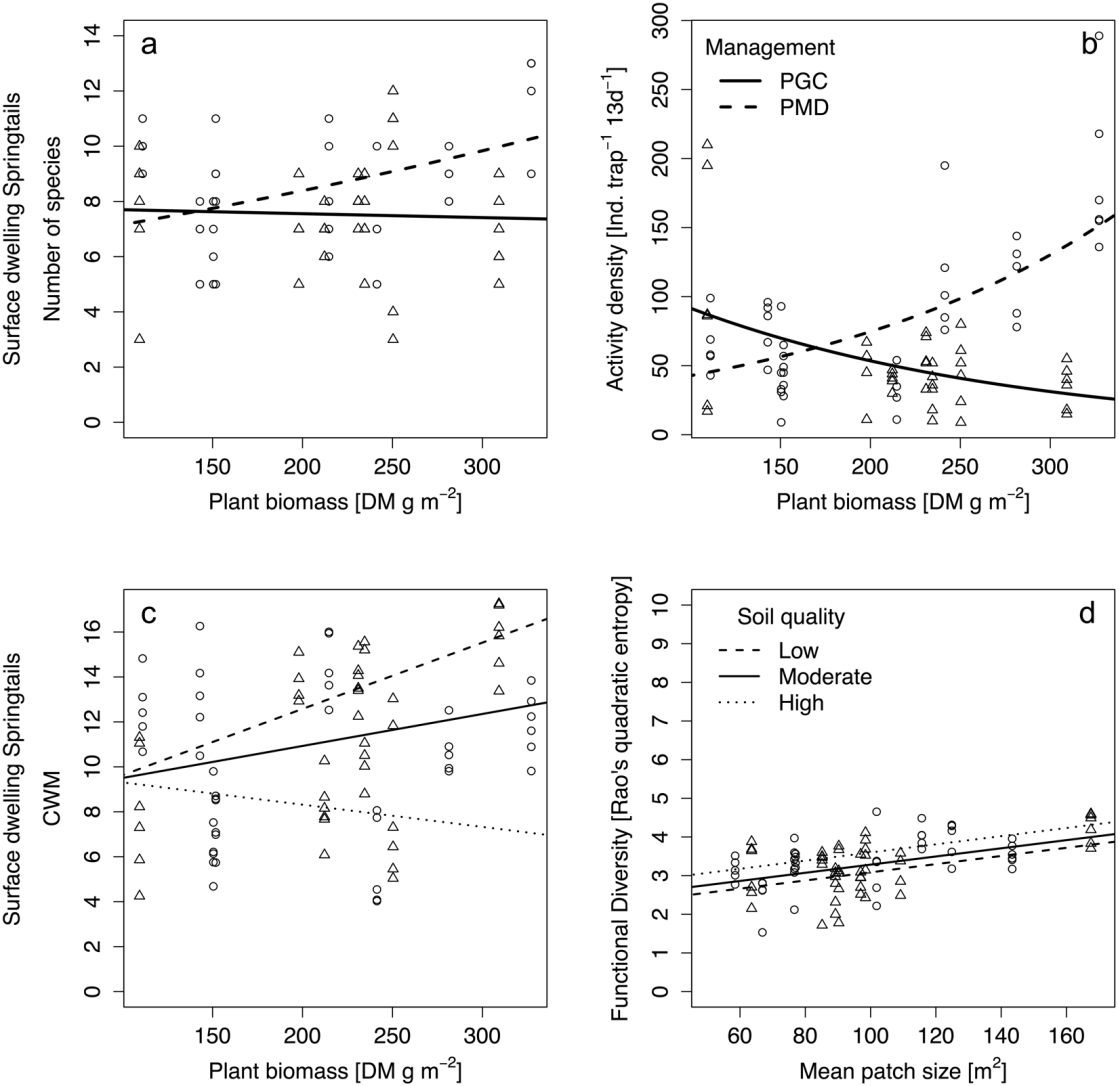
Surface springtail species numbers (**a**), activity density (**b**), community weighted morphological trait value (CWM; (**c**) and functional diversity (**d**) in response to plant biomass, inter-row soil management (PGC…permanent green cover, PMD…periodical mechanical disturbance) or landscape mean patch size (MPS). O…PGC, Δ…PMD, Pitfall trapping, N = 6.

Surface-dwelling springtails species richness was best explained by plant biomass and soil management (R^2^_adj._ = 0.43; Table 2; Fig. 3a). Under PGC, plant biomass had a small effect on springtail species richness. However, increasing plant biomass had a negative effect on springtail species richness. Soil management effect was negligible when plant biomass was low. Nevertheless, the effect of plant biomass was higher under PMD and increased the richness of surface springtails noticeably, with high plant biomass.

Surface-dwelling springtail activity was affected by plant biomass and management (R^2^_m_ = NA; Table 2; Fig. 3b). Similar to the results for surface-dwelling springtail species richness, the effect of plant biomass differed noticeably between the management regimes. While density was higher under PGC than under PMD, when plant biomass was low, increasing plant biomass decreased springtail activity under PGC considerably, whereas the response was positive under PMD.

Surface springtail CWM was affected by plant biomass, soil quality, the their interactions (R^2^_m_ = 0.39; Table 2, Fig. 3c). The CWM was similar across soil quality levels when there was little plant biomass in the vineyard interrow. While CWM increased with plant biomass in sites with moderate soil quality, the effect size was almost twofold on sites with low soil quality. By contrast, on sites with high soil quality increasing plant biomass reduced the CWM.

Functional diversity of surface-dwelling springtails was also influenced by soil quality and mean patch size in the surrounding landscape (MPS; R^2^_m_ = 0.30, Table 2; Fig. 3d). Functional diversity increased with increasing MPS, however, the effect was marginal. Also, the effect of soil quality on functional diversity surface-dwelling springtails was small.

### Litter decomposition

Litter decomposition rate (k) was 0.017 ± 0.009 for PMD and 0.016 ± 0.007 for PGC, stabilisation factor (S) was 0.442 ± 0.067 for PMD and 0.454 ± 0.072 for PGC. Decomposition rate was influenced by plant biomass, soil management and soil quality (R^2^_m_ = 0.06; Table 1). Stabilization factor was influenced by plant biomass, management, soil quality and their interactions (R^2^_m_ = 0.06; Table 1). However, R^2^ obtained with these models were very low.

## Discussion

Overall, our results showed that site factors such as plant biomass and soil quality were more decisive for earthworms and springtails than inter-row soil management. The surrounding landscape structure appeared to affect only the functional diversity of surface dwelling springtails but had no impact on earthworms or soil-dwelling springtails. Litter decomposition remained little affected by the investigated factors and was not related to earthworm or springtail parameters.

We observed a higher species richness of endogeic earthworms in periodically mechanically disturbed (PMD), compared to permanent green covered (PGC) inter-rows, while anecic species number remained unaffected. This is in contrast to other studies showing sensitivity of earthworm species richness to mechanical vineyard inter-row management^8–10^. However, the unresponsiveness of anecic species numbers is in line with reports showing a higher tolerance to soil tillage, compared to endogeic species because of a better ability to escape from disturbance through their vertical burrows^8^. We interpret our findings as follows. First, earthworm populations could have recovered quickly after the last tillage event in the PMD vineyards^9^ as in our study, the last tillage events in the PMD plots had occurred 1–5 years before sampling. Second, we often found an interaction between plant parameters and earthworm species numbers indicating that plants established after tillage compensated for the detrimental mechanical effects. Indeed, plant diversity has been shown to correlate positively with earthworm biomass and density^5,53,54^. Third, tilling in our vineyards is performed at an average depth of 17 cm mainly during dry soil conditions when earthworms might be active in deeper soil horizons and therefore not affected. The lack of epigeic earthworm species in our vineyards reflects the sensitivity of this group to frequent disturbances (e.g., pruning activities, tractor traffic during pesticide applications) taking place in vineyards during the whole season^8,11,55^. Therefore, we conclude, that potential negative effects of soil tillage on earthworms appeared to be levelled out in the next season provided that inter-rows are then covered with vegetation.

Across treatments, earthworm species richness, density and biomass, as well as anecic species richness was positively affected by soil quality underlining their role as bioindicators for soil quality^56^. It was interesting to see that in sites with high soil quality the density of anecic earthworms decreased with increasing plant biomass as this ecological group usually feeds on plant litter at the soil surface^57^. As earthworms differentially respond to plant species this finding suggests that plant nutritional quality at high soil quality with more grasses and less legumes, was not as attractive for earthworms as under lower soil quality^5,7,54,58^.

The finding that densities of soil dwelling springtails were lower in the soils of PGC compared to PMD vineyards concurs with other studies conducted in vineyards^13^. Because in the current study both management regimes had plant cover during sampling, we assume that these effects are mainly due to soil compaction rather than habitat disturbance^59^ or the lacking vegetation cover under PMD^13^. This interpretation is supported by the results of CWM in the soil samples, which were lower in PMD vineyards. The CWM value indicates which life-form dominates a springtail community. Small springtails with reduced pigmentation and furca, absence of ocelli and small antennae, indicated by a small morphological trait value, are more adapted to life in soil where it is difficult to disperse. In contrast larger and pigmented species, with eyes, longer antennae and fully developed furca are better adapted to favourable conditions for dispersal on the soil surface^60,61^. Hence, our results suggest that PGC management provided a less favourable habitat for larger euedaphic species. In support it was reported that springtail life-form types differ in their response to tillage depending on soil texture, i.e., soil dwelling species were harmed by tillage in loamy soils, while surface dwelling species remained relatively unaffected^59^. Our findings for a 54%-increased activity density and a 3.5%-increased species richness of surface dwelling springtails under PMD compared to PGC seemed to disagree with those studies. A likely explanation for these findings can be found in the interaction between tillage and plant biomass. The activity of surface dwelling springtails increased under PMD and decreased under PGC when plant biomass increased. There are two possible explanations for this outcome. First, it could be a consequence of the manifold functions of plant biomass which could serve as a source of nutrients, creating a favourable microclimate and function as a refuge against predation for edaphic springtails^13,62^. Contrary, plant cover also hinders rapid movement of springtails increasing the likelihood of capture^63^. Predators like spiders and carabids also benefit from vegetation cover in vineyard inter-rows^64,65^. It is possible that there was a higher presence of predators in PGC, compared to PMD, where every second row represented a disturbed habitat. However, we did not evaluate the direct relationship between springtails and predators in the current study. Second, in our case the relation between springtail activity, tillage and plant biomass might reflect the microclimate created by the litter layer in PGC, which altered the aboveground springtail community composition and density^66,67^. Additionally, plant biomass promoted larger surface-dwelling species in sites with low and moderate soil quality, while in vineyards of high soil quality, plant biomass production promoted smaller soil-dwelling species. In this context, soil-dwelling species affect the soil organic matter decomposition, whereas surface-dwelling species also feed on aboveground plant material and affect the dynamics of the first stages of litter decomposition^68^. Interestingly, CWM and functional diversity of soil dwelling community showed the opposite response to soil quality and plant biomass. In high-quality soils, plant biomass increased springtail functional diversity, probably due to enhanced food resources available for springtails^66^. Species richness of soil dwelling springtails was only marginally affected by site conditions and plant biomass, a finding which is in line with the results from other vineyards^13^ and arable fields^23^.

The only soil biota parameter influenced by the surrounding landscape was the functional diversity of surface dwelling springtails. Springtail functional diversity increased with increasing mean patch size (MPS), where a high MPS indicates low fragmentation and low heterogeneity of the landscape. This is in contrast with the general assumption that heterogeneous landscapes positively affect springtail density and species richness^52,69^. Reasons for these contrasting patterns could be that different landscape matrices and land use histories were studied: agro-forest mosaics^31,49^ versus viticultural landscapes in the current study. Another reason could be that the very small-structured viticultural landscapes of the study regions precluded the build-up of functionally diverse springtail communities or the 2-m high grapevine trellis negatively affected springtail dispersal. The absence of landscape effects on earthworms suggests little connection between non-crop habitats and vineyards. However, to draw a more general conclusion on the influence of viticultural landscapes on earthworms more detailed analyses including earthworm tracking would be necessary.

Litter decomposition was unaffected by soil management and was only weakly predicted by the other site and landscape parameter we assessed in this study. Because the mesh size of the decomposition bags excluded larger springtails and all earthworms the contribution of smaller springtails to litter decomposition appeared to be marginal. Since vineyards are always treated with several pesticides a lack of response of decomposition could also mean that pesticide effects on non-target soil biota overrode potential tillage or landscape effects on litter decomposition^38,70,71^. Clearly, it would need more specific experimental approaches to disentangle underlying interrelationships.

In conclusion, our study suggests that response patterns of soil biota to soil management in vineyards differ from those known from arable agroecosystems, where negative effects of intensification on soil biota are common^72^. Earthworms and springtails in vineyard inter-rows differed in their sensitivity to soil disturbance and were modified by plant biomass and soil quality. While the size and diversity of earthworm populations were little affected by soil tillage, mainly larger-sized surface and soil dwelling springtails were reduced in vineyards with permanent green cover. Our results suggest that effects of inter-row tillage on the diversity and functionality of soil biota could be compensated by establishing a plant cover during the rest of the year. We conclude that soil biota in vineyards situated in heterogeneous landscapes are mainly influenced by site and management factors.

## Methods

### Site description and study design

The study sites are located in neighbouring wine regions Carnuntum (48° 4′N, 16° 47′E, province of Lower Austria) and Leithaberg (47° 54′N, 16° 41′E, province of Burgenland), about 40 km south east of Vienna, Austria. In these regions, wine cultivation has a long tradition dating back to the Roman Empire in the 1^st^ century B.C. Typically, vineyards in these regions are small (0.4–1 ha) belonging to numerous wine growers. Vineyards are interspersed within a more or less heterogeneous landscape adjoined by arable crops and several non-crop landscape elements such as fallows, forests or hedges. The climate in the regions is continental with annual average temperature of 11.5 °C and annual rainfall of 530 mm (between 1990 and 2004; weather data for the city of Bruck/Leitha which is located in the centre of the study region). In the study region the most frequent soil types are Chernozems and Cambisols. Vineyards are cropped with red or white grape varieties cultivated in a vertical trellising system with within-row grapevine distance of 0.75–1.30 m and inter-row distance varying between 2.15–3.00 m.

Within these regions we selected 16 commercial vineyards along an altitudinal range of 120–260 m a.s.l. In 8 vineyards the inter-rows had permanent green cover (PGC) and in 8 vineyards every second inter-row was periodically mechanically disturbed (PMD). Both management regimes were practiced for at least 5 years. Management data was collected with a questionnaire among the participating wine growers (Supplementary Table S3). Inter-row vegetation originates from a cover crop seed mixture or from the spontaneous vegetation. Vegetation was mulched 1 to 5 times per year. Application of organic (shredded pruning material, compost, green manure) or inorganic nitrogen-phosphorous-potassium-fertilizers, was done according to good wine-growers practice. Among the wine-growers, three were certified organic farmers; the remaining vineyards were managed conventionally. As inter-row soil management does not differ between conventional or organic vineyards, we included both farming practices.

At each vineyard, we selected one inter-row sampling area (approximately 2 × 40 m) in the middle of the vineyard; this area had at least 5 m distance to the vineyard edge. In PMD sites, samples were taken in the inter-rows that had been cultivated in the previous year.

### Sampling and measurements

#### Earthworm sampling

From April 13–21, 2015 we sampled earthworm communities by hand-sorting of 6 soil blocks (20 × 20 × 25 cm, L × W × H) along a transect in the middle of the vineyard inter-row; distance between excavated soil blocks was 5–7 m. Earthworms were conserved in 4% formaldehyde and identified at the Station Biologique of the University of Rennes, Paimpont, France.

In the laboratory, earthworms were identified to the species level, counted, weighed, and categorized into the ecological groups epigeics, anecics and endogeics according to Bouché^73,74^.

#### Springtail sampling

Soil dwelling springtail communities were sampled by taking 4 soil cores (5.5 × 5.5 × 10 cm, L × W × H) between April 13–21, 2015 in about 50 cm distance of the installed pitfall traps at each vineyard site. Soil samples were immediately sealed in plastic bags and transported in a cooling box to the laboratory for extraction using a Berlese-Tullgren extractor for 4 days. In the extractor, the animals were collected in plastic jars filled with 10% benzoic acid solution. After extraction, samples were washed with tap water in a 0.063 mm sieve in order to clear from adherent soil particles and then stored in 80% ethanol alcohol. Springtails were sorted and determined to species level, except of *Mesaphorura* sp, which were determined to genus level only. Identification of springtail was done using several determination keys^75–83^.

Surface dwelling springtail species were collected using six pitfall traps (diameter 17 mm, length 60 mm) installed on April 21, 2015 at each vineyard site^84^. Traps were inserted with about 6 m distance to each other along a transect in the middle of inter-rows and filled with ethylene glycol and a drop of odourless detergent. After an exposure of 13 days in the field, the traps were removed, sorted for springtails and determined to species level and counted. The number of trapped springtails during the sampling period is referred to as activity density.

Additionally to the taxonomic identification, springtails were assigned morphological trait values^61,85^. The trait value attributes a measurement or a score to each of the following traits: number of ocelli, body size, body pigmentation level and pattern, presence of modified hairs or scales, furca development and antennae length (Supplementary Table S4). Traits were extracted from literature used for identification. For body size we chose the maximum value found in the most recent literature. When there was more than one specification for an attribute, we used the dominant expression of the respective species in our samples as reference (e.g. *Proisotoma notabilis* can have 2 + 2 to 5 + 5 ommatidia (compound eyes)^80^, however we counted 4 + 4 ommatidia on the individuals in our samples).

We used the number of species and densities as references to taxonomic diversity and calculated the community weighted morphological trait value (CWM)^85^, as an index of the dominant trait value in the springtail community. Further we calculated the springtail functional diversity (FD) by computing the Rao’s quadratic entropy using the “FD” package^86^ in the software R^87^.

#### Litter decomposition

Litter decomposition was assessed using the teabag index (TBI)^88^. Therefore, we buried 10 pairs of green tea and rooibos teabags (EAN: 87 22700 05552 5 and EAN: 87 22700 18843 8, for green and rooibos tea, respectively; Lipton Tea, Washington St, USA) between April 13–21, 2015. Teabags were inserted at a soil depth of 8 cm along a transect in the middle of the inter-row; distance between teabags was about 5–7 m. The mesh size of the tea bags of 0.25 mm allows micro-, and mesofauna to enter, but excludes macrofauna^89^. After approx. 90 days the teabags were recovered, dried, and weighed. About 15% of the teabags were destroyed by mice or farming machinery during exposition in the field. The TBI consists of the decomposition rate (k) and the stabilisation factor (S); k refers to the first phase of decomposition; S accounts for the labile fraction of the material which is not decomposed, but stabilized. We used the average S value for calculating k.

#### Vegetation and soil quality parameters

Vegetation cover of each vascular plant species was measured once in April and June 2015 on four 1 × 1 m plots located in the centre of each vineyard inter-row. Cover percentage of each vascular plant species was estimated following the scale of Londo^90^. For statistical analysis, we calculated the plant cover percentages of legumes, grasses and herbs and the Shannon diversity index^91^. In May, aboveground plant biomass production was assessed by cutting all vegetation on four 1 × 0.5 m plots, drying at 60 °C for 48 hours and weighing the dry matter (Table 3).

**Table 3:** Overview of site, vegetation and landscape variables used for the statistical analyses. Means ± SD.

Parameter		Periodical mechanical disturbance (PMD)	Permanent green cover (PGC)
Last soil disturbance (years ago, mean)		2.5	25
Tillage depth (cm)		17 ± 3	n.a.
**Site parameters**			
Soil quality (field index)		49 ± 1	40 ± 8
**Vegetation**			
Biomass (DM g m^−2^)		101.3 ± 38.1	109.2 ± 33.0
Plant cover (%)		73.4 ± 18.5	85.5 ± 8.4
Legume cover (%)		15.8 ± 12.7	7.7 ± 8.0
Herb cover (%)		43.6 ± 18.7	39.7 ± 15.9
Grass cover (%)		40.6 ± 21.8	52.6 ± 10.8
Species richness		13 ± 2	13 ± 3
**Landscape**			
Distance to next semi-natural element (m)		20.8 ± 15.6	
Distance to next crop (m)		1.6 ± 0.3	
Mean patch size (MPS; m^2^)		97.3 ± 29.1	
Diversity (Shannon)		1.6 ± 0.3	

n.a. not applicable.

As a proxy of soil quality in the vineyards we used the Austrian soil field index in our analysis (Table 3). This soil field index is assigned to all agriculturally used parcels of land and is used to calculate agricultural land taxes in Austria. It represents the natural yield capacity of a field in relation to the highest yielding capacity of the country; values from 0 to 100 points, where 100 points stands for the highest yield capacity. The soil index integrates soil type, humus content, soil depth, soil texture, bulk density, soil structure, lime content, gleying and soil congregation as well as climatic and hydrologial properties and the slope of the site^92^.

#### Landscape parameters

Field mapping was done in July 2015. As a reference we used the official Austrian land utilization map from 2012 (Integriertes Verwaltungs- und Kontrollsystem, INVEKOS). Landscape elements within a 750 m-radius were classified into habitat types according to CORINE Land Cover and EUNIS Habitat Classification^93,94^. Habitat types were then clustered into two levels of classification. The first level classification is composed by semi-natural elements (SNE: hedges, tree rows, grass stripes, natural grassland, pasture, fallow, heathland, wetland and woodlots), viticulture, other agricultural land (annual crops), open land, wood, water entities, and artificial/constructed entities (urban area, buildings and roads). The more detailed landscape classification was used for calculating the Shannon diversity Index which represents landscape composition, the mean patch size (MPS), which respresents landscape configuration and the distances to closest SNE and crop. Mapping and analysis was done using the programs ArcGis 10.2.1^95^, QGis 2.8.1, FRAGSTATS 4.2^96^ and CHLOE2012^97^.

### Statistical analysis

For earthworm analyses data from all 96 soil excavations were used; for collembolan analyses we used the data from 64 soil cores (i.e. 4 soil cores for each vineyard) and 89 pitfall traps (usually 6 traps per vineyard, except 7 traps that were filled with soil during exposure in the field).

Because of Poisson error distributions of the response variables, we fitted generalized linear mixed models (GLMM) for our nested experimental design to analyse the effects of management and soil quality on earthworms, collembolan and decomposition and how these effects are altered by site and landscape variables. Vineyard was used as a random factor in the models. Further, we used model selection as an alternative to traditional hypothesis testing^64,98^. Prior to fitting GLMMs we excluded several explanatory variables following a selection procedure based on scatterplots between responsive and explanatory variables: first we selected the site and landscape variables correlating highest (r > 0.18) with the response variables. Provided there was linear correlation (r > 0.3) with vineyard management or soil quality, we removed these variables from the analysis (e.g., farming system organic or conventional). Remaining variables were then tested on co-linearity with subsequent exclusion of one of the correlating variables from the analysis. As a result, plot parameters (plant richness, plant cover, grass, herb cover) and landscape parameters (SNE, viticulture, other agriculture, open land, wood, water entities, artificial/constructed entities, distances to next crop and Shannon landscape diversity) were excluded from the GLMM analysis. Afterwards, we built two sets of models (in total 46 models), one with management and one with soil quality as fixed factors with the selected plant (plant biomass, legume cover) and landscape variables (MPS, distance to next SNE) as covariables and vineyard as random effect variable. To be able to test for effects of different soil quality levels we considered plots with a field index < 23 as low, plots with a field index > 67 as high; the remaining plots were considered with medium soil quality.

Response variables were square-rooted or log transformed to account for non-normal distribution when necessary. The analyses were performed using the “lme4” package^99^ and the “glmmADMB” package^100^ in the software R^87^.

For model selection we used the Akaike Information Criterion corrected for small sample sizes (AIC_c_), a measure of the quality of a statistical model. The most parsimonious model was considered the best model to describe the significant effect on the response variable. Models where AIC_c_ was less than 2 units above the AIC_c min_ were considered identical good fits as the best model. In such a case the most complex model (number of variables, interaction, random factor) was plotted and used for further interpretation. In plots containing two continuous variables, soil biota response was plotted against one variable at the 5%, 50% and 95% levels of the second variable. Model assumptions were verified using diagnostic plots and the Package “DHARMa”^101^.

We calculated the R^2^ for the best models to account for the variability explained by mixed models with Gaussian or Poisson error distributions^102^. Though this approach cannot be applied to models with a negative binomial error distribution, it allows for two components of R^2^ to be calculated: (i) a marginal R^2^ (R^2^_m_) that only includes the variance explained by fixed effects; and (ii) a conditional R^2^ (R^2^_c_) that represents the variance explained by both fixed and random effects. In case of two or more best fitting models, R^2^_m_ and R^2^_c_ were calculated for the more complex model. For models without random effects we calculated the adjusted R^2^.

## Electronic supplementary material

Supplementary Information
