# Author Correction: Soil biota in vineyards are more influenced by plants and soil quality than by tillage intensity or the surrounding landscape

**DOI:** 10.1038/s41598-018-24797-y

**Published:** 2018-04-19

**Authors:** Jacob Buchholz, Pascal Querner, Daniel Paredes, Thomas Bauer, Peter Strauss, Muriel Guernion, Jennifer Scimia, Daniel Cluzeau, Françoise Burel, Sophie Kratschmer, Silvia Winter, Martin Potthoff, Johann G. Zaller

**Affiliations:** 1 Institute of Zoology, University of Natural Resources and Life Sciences Vienna (BOKU), Gregor Mendel Straße 33, 1180 Vienna, Austria; 2 Grupo de Protección Vegetal, Departamento de Protección Ambiental, Estación Experimental de Zaidín, CSIC, Profesor Albareda n◦1, 18008 Granada, Spain; 3 Institute for Land and Water Management Research, Austrian Federal Agency for Water Management, Pollnbergstraße 1, A-3252 Petzenkirchen, Austria; 4 Université de Rennes I, OSUR, UMR CNRS 6553 ‘EcoBio’, Station Biologique de Paimpont, 35380 Paimpont, France; 5 Université de Rennes I, OSUR, UMR CNRS 6553 ‘EcoBio’, Avenue du Général Leclerc Campus de Beaulieu, F-35042 Rennes, Cedex France; 6 Institute of Integrative Nature Conservation Research, University of Natural Resources and Life Sciences Vienna, Gregor Mendel Straße 33, 1180 Vienna, Austria; 7 Centre of Biodiversity and Sustainable Land Use (CBL), University of Göttingen, Grisebachstraße 6, 37077 Göttingen, Germany

Correction to: *Scientific Reports* 10.1038/s41598-017-17601-w, published online 12 December 2017

The Supplementary Information file was omitted from the original version of this Article. This has been corrected in the HTML version of the Article; the PDF version was correct at time of publication.

