# Supplementary material for: Soil biota in vineyards are more influenced by plants and soil quality than by tillage intensity or the surrounding landscape
Source: Sci Rep. 2017 Dec 12;7:17445. doi: 10.1038/s41598-017-17601-w (PMC5727173; doi:10.1038/s41598-017-17601-w)
Supplement: Supplementary file 1 — Supplementary Information [file 41598_2017_17601_MOESM1_ESM.pdf]

# SUPPLEMENTARY MATERIAL

## Manuscript: Soil biota in vineyards are more influenced by plants and soil quality than by tillage intensity or the surrounding landscape

Jacob Buchholz, Pascal Querner, Daniel Paredes, Thomas Bauer, Peter Strauss, Muriel Guernion, Jennifer Scimia, Daniel Cluzeau, Françoise Burel, Sophie Kratschmer, Silvia Winter, Martin Potthoff, Johann G. Zaller

Supplementary Table S1. Earthworm species and mean ± SD density $m^{-2}$ in vineyards where inter-rows have been periodically mechanically disturbed (PMD) or left with permanent green cover (PGC).

| Earthworm functional groups/species | Density $m^{-2}$ | |
|---|---|---|
| | PMD | PGC |
| Anecics | | |
| *Aporrectodea caliginosa* (anecic) (Savigny, 1826) | 1.0 ± 5.1 | 0.5 ± 3.6 |
| *Aporrectodea* sp.* | 0.5 ± 3.6 | 0.5 ± 3.6 |
| *Lumbricus terrestris (L.)* | 5.2 ± 19.3 | 0 |
| *Lumbricus* sp. (juv.)* | 12.0 ± 23.6 | 0 |
| Morphotype anecic* | 18.8 ± 41.7 | 11.5 ± 24.7 |
| Endogeics | | |
| *Aporrectodea caliginosa* (endogeic) (Savigny, 1826) | 78.6 ± 89.6 | 24.5 ± 63.7 |
| *Aporrectodea rosea* (Savigny, 1826) | 31.3 ± 42.4 | 59.4 ± 97.0 |
| Morphotype endogeic* | 16.7 ± 21.5 | 8.3 ± 14.9 |
| *Proctodrilus antipai* (Michaelsen, 1891) | 6.8 ± 15.2 | 0 |
| *Octolasion lacteum* (Örley, 1885) | 2.6 ± 9.3 | 5.2 ± 15.4 |
| *Allolobophora chlorotica* (Savigny, 1826) | 6.3 ± 23.9 | 0.5 ± 3.6 |
| *Aporrectodea* sp. (juv.)* | 0 | 1.0 ± 5.1 |
| *Octolasion* sp. (juv.)* | 2.1 ± 7.0 | 0 |
| not identified | 0 | 0.5 ± 3.6 |

* these specimens could not be identified to the species level.

Supplementary Table S2. Springtail species and mean ± SD densities in soil samples (5.5 x 5.5 x 10 cm) and mean ± SD activity density in the pitfall traps in vineyards where inter-rows have been periodically mechanically disturbed (PMD) or left with permanent green cover (PGC).

| Springtail species | Soil samples | | Pitfall traps | |
|---|---|---|---|---|
| | PMD | PGC | PMD | PGC |
| *Axenyllodes bayeri* (Kseneman 1935) | 693.0 ± 1917.9 | 1365.4 ± 4968.7 | - | - |
| *Ceratophysella bengtssoni* (Ågren 1904) | 0 | 10.3 ± 58.5 | - | - |
| *Ceratophysella quinqisetosa* cf. (Gisin 1958) | 868.9 ± 4337.6 | 1034.4 ± 2982.9 | - | - |
| *Ceratophysella sigillata* (Uzel 1891) | 0 | 10.34 ± 58.51 | - | - |
| *Ceratophysella succinea* (Gisin 1949) | 41.4 ± 234.1 | 186.2 ± 365.9 | 0.6 ± 3.7 | 1.1 ± 2.1 |
| *Cryptopygus bipunctatus* (Axelson 1903) | 3796.2 ± 13678.5 | 0 | - | - |
| *Cryptopygus orientalis* (Stach 1947) | 31.0 ± 98.0 | 10.3 ± 58.5 | - | - |
| *Cyphoderus albinus* (Nicolet 1842) | 10.3 ± 58.5 | 124.1 ± 383.0 | 0.2 ± 0.8 | 0.2 ± 0.9 |
| *Cyphoderus bidenticulatus* (Parona 1888) | 0 | 10.3 ± 58.5 | 0.1 ± 0.2 | 0 |
| *Desoria fennica cf.* (Reuter 1895) | 206.9 ± 574.9 | 10.3 ± 58.5 | 0 | 0.1 ± 0.2 |
| *Desoria olivacea* (Tullberg 1871) | 206.9 ± 473.7 | 51.7 ± 148.3 | 4.7 ± 8.5 | 1.7 ± 2.2 |
| *Desoria* sp. | 10.3 ± 58.5 | 0 | - | - |
| *Entomobrya handschini* (Stach 1922) | 10.3 ± 58.5 | 0 | 1.8 ± 1.9 | 4.8 ± 4.5 |
| *Entomobrya marginata* (Tullberg 1871) | 0 | 10.3 ± 58.5 | 2.7 ± 6.1 | 0.5 ± 1.1 |
| *Entomobrya multifasciata* (Tullberg 1871) | - | - | 0 | 0.1 ± 0.2 |
| *Folsomia candida* (Willem 1902) | 113.8 ± 381.7 | 0 | - | - |
| *Folsomides parvulus* (Stach 1922) | 175.8 ± 468.0 | 682.7 ± 2436.6 | - | - |
| *Heteromurus major* (Moniez 1889) | 300.0 ± 816.7 | 103.4 ± 471.4 | 17.4 ± 17.3 | 13.2 ± 15.9 |
| *Heteromurus nitidus* (Templeton 1835) | 0 | 20.7 ± 117.0 | 1.0 ± 1.2 | 0.9 ± 2.0 |
| *Heterosminthurus claviger* (Gisin 1958) | - | - | 0.1 ± 0.3 | 0 |
| *Hypogastrura sahlbergi* (Reuter 1895) | 0 | 31.0 ± 175.5 | - | - |
| *Isotoma viridis* (Bourlet 1839) | 993.0 ± 1275.1 | 1965.3 ± 3615.1 | 29.0 ± 25.0 | 17.7 ± 25.3 |
| *Isotomodes productus* (Axelson 1906) | 113.8 ± 643.6 | 0 | - | - |
| *Isotomurus* sp. | 82.8 ± 265.9 | 0 | - | - |
| *Lepidocyrtus cyaneus* (Tullberg 1871) | 41.4 ± 111.2 | 0 | 13.2 ± 14.6 | 2.2 ± 2.9 |

| | | | | |
|---|---|---|---|---|
| *Lepidocyrtus lanuginosus* (Gmelin 1788) | 10.3 ± 58.5 | 10.3 ± 58.5 | 0.7 ± 2.0 | 0.1 ± 0.6 |
| *Lepidocyrtus lignorum* (Fabricius 1775) | 62.1 ± 244.2 | 10.3 ± 58.5 | 0 | 0.2 ± 0.7 |
| *Lepidocyrtus paradoxus* (Uzel 1891) | 0 | 51.7 ± 148.3 | 1.1 ± 2.0 | 1.5 ± 2.7 |
| *Mesaphurora* sp. | 7023.4 ± 7871.0 | 9464.5 ± 8822.9 | - | - |
| *Metaphorura affinis* (Börner 1902) | 1168.8 ± 3947.9 | 672.3 ± 1413.1 | 0 | 0.1 ± 0.2 |
| *Metaphorura riozoi* (Castaño-Meneses, Palacios-Vargas & Traser 2000) | 848.2 ± 2695.5 | 1117.1 ± 2776.7 | - | - |
| *Micranurida pygmaea* (Börner 1901) | 0 | 920.6 ± 2091.3 | - | - |
| *Neotullbergia ramicuspis* (Gisin 1953) | 796.5 ± 1255.1 | 134.5 ± 384.0 | - | - |
| *Orchesella cincta* (Linnaeus 1758) | 0 | 41.4 ± 139.4 | 5.8 ± 7.8 | 5.4 ± 6.5 |
| *Orchesella pannonica* (Stach 1960) | - | - | 0.1 ± 0.2 | 0.1 ± 0.2 |
| *Parisotoma notabilis* (Schäffer 1896) | 10167.9 ± 16787.9 | 17077.5 ± 25851.0 | 1.2 ± 2.7 | 2.3 ± 6.5 |
| *Pogonognathellus flavescens* (Tullberg 1871) | - | - | 0.1 ± 0.3 | 0.1 ± 0.5 |
| *Proisotoma* sp. | 41.4 ± 183.2 | 0 | - | - |
| *Protaphorura armata* (Tullberg 1869) | 130083.0 ± 9966.0 | 868.9 ± 1621.0 | - | - |
| *Protaphorura subfimata* (Thibaud & Christian 1986) | 0 | 2037.7 ± 6840.1 | - | - |
| *Pseudachorutella asigillata* (Börner 1901) | 0 | 10.3 ± 58.5 | 0 | 0.2 ± 0.6 |
| *Pseudachorutes dubius* (Krausbauer 1898) | 0 | 10.3 ± 58.5 | - | - |
| *Pseudachorutes parvulus* (Börner 1901) | 113.8 ± 472.7 | 62.1 ± 155.9 | 0.1 ± 0.2 | 0.6 ± 1.9 |
| *Pseudosinella alba* (Packard 1873) | 206.9 ± 509.7 | 51.7 ± 170.4 | 0.8 ± 2.4 | 0.4 ± 1.2 |
| *Pseudosinella duodecimpunctata* (Denis 1931) | 10.3 ± 58.5 | 0 | - | - |
| *Pseudosinella octopunctata* (Görner 1901) | 10.3 ± 58.5 | 0 | - | - |
| *Pseudosinella petterseni* (Börner 1901) | 62.1 ± 196.1 | 0 | 0.1 ± 0.2 | 0 |
| *Pseudosinella sexoculata* (Schött 1902) | 0 | 10.3 ± 58.5 | 0.3 ± 0.7 | 0.1 ± 0.3 |

Table 6 (continued)

| | | | | |
|---|---|---|---|---|
| *Seira* spp. | - | - | 0 | 0.1 ± 0.2 |
| *Sminthurinus aureus* (Lubbock 1862) | 548.2 ± 1062.2 | 103.4 ± 213.3 | 4.0 ± 4.1 | 1.5 ± 1.8 |
| *Sminthurus multipunctatus* (Schäffer 1896) | - | - | 0.1 ± 0.4 | 0.1 ± 0.2 |
| *Sminthurus viridis* (Linnaeus 1758) | 10.3 ± 58.5 | 0 | 0.1 ± 0.2 | 0 |

| | | | | |
|---|---|---|---|---|
| *Sphaeridia pumilis* (Krausbauer 1898) | 817.2 ± 2224.4 | 103.4 ± 319.5 | 0.7 ± 0.9 | 0.7 ± 1.2 |
| *Stenaphorurella denisi* (Bagnall 1935) | 10.3 ± 58.5 | 341.3 ± 1084.8 | - | - |
| *Stenaphorura quadrispina* (Börner 1901) | 0 | 72.4 ± 311.6 | - | - |
| *Tomocerus vulgaris* (Tullberg 1871) | - | | 0 | 0.1 ± 0.2 |
| *Willemia buddenbrocki* (Hüther 1959) | 20.7 ± 81.4 | 51.7 ± 292.6 | - | - |
| *Willemia intermedia* (Mills 1934) | 93.1 ± 192.4 | 268.9 ± 1110.4 | - | - |
| *Willemia scandinavica* (Stach 1949) | 631.0 ± 1596.0 | 941.3 ± 1664.8 | - | - |
| *Willowsia nigromaculata* (Lubbock 1873) | - | - | 0.1 ± 0.2 | 0 |
| *Xenylla maritima* (Tullberg 1869) | - | - | 0 | 0.1 ± 0.2 |
| not identified | 82.8 ± 237.8 | 72.4 ± 139.0 | - | - |

Supplementary Table S3: Management characteristics of the study vineyards where inter-rows have been periodically mechanically disturbed (PMD) or left with permanent green cover (PGC).

| Management | Periodical mechanical disturbance (PMD) | Permanent green cover (PGC) |
|---|---|---|
| Farming type | 1 organic, 7 conventional | 2 organic, 6 conventional |
| Last disturbance event | 1- 4 years | 5-45 years |
| Soil cultivation machinery | chisel plow (depth: 15-30 cm), harrow (depth 3-15 cm), deep loosening (25-40 cm) | N.A. |
| Inter-row vegetation | Specific seed mixtures for vineyards (legumes, herbs), spontaneous vegetation | |
| Fertilization | Shredded pruning material, green manure, compost, pomace, inorganic NPK | |

Supplement Table S4: Morphological traits of springtails adapted from Vandewalle et al., (2010) and Martins da Silva et al., (2015b)

| Traits | Data type | Attribute type |
|---|---|---|
| Number of ocelli | Ordinal | 0-8 |
| Body size | Quantitative | in mm |
| Pigmentation level | Ordinal | 0 = white, 1 = lightly pigmented, 2 = intensly pigmented |
| Pigmentation pattern | Binary | 0 = absent, 1 = present |
| Hairs/scales | Binary | 0 = absent, 1 = present |
| Furca | Ordinal | 0 = absent, 1 = reduced/short, 2 = fully developed |
| Antenna/body ratio | Ordinal | 0 = $X \leq 0.5$; 1 = $0.5 < X \leq 1$; 2 = $X > 1$ |
